# Falls in institutionalized elderly with and without cognitive decline A study of some factors

**DOI:** 10.1590/1980-57642018dn13-010014

**Published:** 2019

**Authors:** Cristina Lavareda Baixinho, Maria dos Anjos Dixe, Carla Madeira, Sílvia Alves, Maria Adriana Henriques

**Affiliations:** 1 Escola Superior de Enfermagem de Lisboa, Unidade de Investigação & Desenvolvimento em Enfermagem, Lisboa, Portugal.; 2 Center for Innovative Care and Health Technology - CiTheCare, Portugal.; 3 Escola Superior de Saúde, Instituto Politécnico de Leiria, Portugal.; 4 Enfermeira Especialista em Enfermagem de Reabilitação, Hospital de Vila Franca de Xira, Portugal.

**Keywords:** nursing, accidental falls, elderly, homes for the aged, cognitive dysfunction, enfermagem, acidentes por quedas, idosos, instituição de longa permanência para idosos, disfunção cognitiva

## Abstract

Falls in long-stay institutions for elderly people have a high prevalence, contributing to the physical and mental deterioration of institutionalized elderly.

**Objective:**

To determine the prevalence of falls among institutionalized elderly with and without cognitive decline, and to characterize the practices and behaviors of those with and without cognitive decline in managing fall risks, and relate them to some factors.

**Methods:**

The present correlational study was carried out with a sample of 204 institutionalized elderly, 50% of whom had cognitive decline.

**Results:**

The elderly with cognitive decline (40.2%) fell less often than those who did not have cognitive decline (42.2%) (p>0.05). Safety practices and behaviors were better in the elderly with cognitive decline (p<0.05). Most of the elderly with cognitive decline who fell took benzodiazepines (65.9%), in contrast with those without cognitive decline (32.2%). It was observed that 81.4% of the elderly without cognitive decline and 43.9% of those with cognitive decline who fell had a performance of over 12 seconds on the Timed Up and Go Test, where differences reached statistical significance in both groups of elderly.

**Conclusion:**

Data collected in the present study further the knowledge on risk factors in the genesis of falls and on the behavior of elderly with and without cognitive decline in maintaining their safety in self-care and accessibility.

## RESUMO.

As quedas nas instituições de longa permanência para idosos apresentam uma elevada prevalência, contribuindo para a deterioração física e mental dos idosos institucionalizados.

**Objetivo:**

Determinar a prevalência de quedas nos idosos institucionalizados com e sem declínio cognitivo; caracterizar as práticas e comportamentos dos idosos com e sem declínio cognitivo na gestão do risco de queda e relacionar com alguns fatores.

**Métodos:**

Este estudo correlacional foi realizado numa amostra de 204 idosos institucionalizados (50% com declínio cognitivo).

**Resultados:**

Os idosos com declínio (40,2%) caíram menos que os que não tem declínio (42,2%) cognitivo (p>0,05). As práticas e comportamentos de segurança são melhores nos idosos com declínio (p<0,05). O maior percentual de idosos com declínio que caem consomem benzodiazepinas (65,9%), o mesmo não se verificando com os idosos sem declínio (32,2%). Verificamos que 81,4% dos idosos sem declínio e 43,9% dos idosos com declínio que caem apresentam perfomance no *Timed Up and Go Test* superior a 12 segundos tendo as diferenças significado estatístico em ambos os grupos de idosos.

**Conclusão:**

Os dados contribuem para um melhor conhecimento dos fatores de risco na génese da queda e das práticas e comportamentos dos idosos com e sem declínio para manterem a sua segurança no autocuidado e a acessibilidade.

Falls in the elderly population are an emerging public health problem, with clear challenges for health policies oriented to this age group. The prevalence of these events may vary according to context, but the consensus is that rates are higher in long-stay institutions for elderly people, with an estimated incidence of between 34% and 67%.^1^^,^ 2

Researchers have attributed this higher rate among institutionalized elderly people to increased levels of dependence, higher numbers of chronic diseases, polymedication,^2^^,^ 3 lack of physical activity,^3^ and greater interactions between intrinsic and extrinsic risk factors.^2^^-^ 4 The nature of the physical spaces, accessibility, and presence of professionals make the institutional setting different from home,^4^ and the suffering caused by the absence of families can by itself represent an extra risk factor.^4^

Cognitive decline has been consistently associated with the risk and occurrence of falls.^5^ In addition, deficits caused by cognitive decline lead to functional decline, with a reduction and/or loss of abilities and increase in unsafe behaviors, which directly or indirectly increases the probability of falls.^5^

A recent literature review sought to identify fall risk factors specific to elderly people with dementia and concluded that there are intrinsic and extrinsic risk factors which are common to elderly without cognitive decline and others that are exclusive to people with dementia. The review found that some risk factors are exclusive to elderly people who show cognitive decline, such as incoherent speech, disruptive behaviors, attention cognitive capacity, cortical alterations, severity of dementia, reduced visual perception, and caregiver overload.^6^

Researchers have noted a lack of consistency in the association of fall risk factors, because studies do not explore all of these. Reports emphasize the need to balance these limitations and to carry out more investigations to define risk attributes.^6^

Practices and behaviors of institutionalized elderly people have been associated with falls. A recent study revealed that elderly people exhibit low adherence to maintaining safe behaviors.^7^ The results showed that the most dependent elderly people were those who had the worst practices and behaviors regarding fall prevention, and that those who used gait assistive devices have better practices of communication and access to physical spaces.^7^ The authors suggested that these differences result from a greater awareness by these elderly of difficulties related to gait and fear of falling.^8^ However, the sample examined in this study comprised institutionalized elderly without cognitive decline.

The authors of the present study agree with other researchers who state that factors related to increased fall risk in people with dementia and cognitive decline are not fully understood, and that this lack of knowledge hinders the development of guidelines aimed at preventing these events.^6^

The objectives of the present study were to determine the prevalence of falls in institutionalized elderly people with and without cognitive decline; to characterize the practices and behaviors of elderly people with and without cognitive decline regarding the management of fall risks; and to investigate the relationship of occurrence of falls, polymedication, use of benzodiazepines, and gender with the practices and behaviors of elderly people with and without cognitive decline.

## METHODS

The sample of the present correlational study comprised institutionalized elderly in two long-stay institutions for this population in the Lisbon and Tagus Valley region in Portugal.

The inclusion criteria were being aged 65 years or older and institutionalized. The following were excluded: fully dependent elderly people, because of the impossibility of applying one of the instruments of data collection, the Scale of Practices and Behaviors of Institutionalized Elderly to Prevent Falls (EPCIPQ, as per its initialism in Portuguese);^7^ and elderly users who only frequented the institution for day-care services.

The application of the Barthel index to evaluate level of dependence, and the Mini-Mental State Examination (MMSE) to determine cognitive condition, allowed the identification of 102 elderly people with, and 102 without, cognitive decline who met the inclusion and exclusion criteria.

The sample was followed up regarding its practices and behaviors by applying the EPCIPQ. The original scale has two dimensions. The first addresses the practices and behaviors of bilateral communication between elderly people and the different professionals at long-stay institutions for elderly people. The second dimension, related to the safety practices and behaviors adopted by elderly people (associated with safe behaviors regarding self-care and accessibility of physical spaces), has 11 items and a score ranging from 11 to 33 points. In the present study, a weighted average was adopted.^1^^-^ 3 Given the typology of elderly people with cognitive decline, only the second dimension was applied in the present study. The instrument has good psychometric characteristics, whether applied through observation or interviews.

The Timed Up and Go Test (TUGT) was used to evaluate gait capacity, quality, and time.^8^ The TUGT is a simple and easy-to-apply test that allows the assessment of mobility and functional balance in elderly people in both communities and institutions.^9^ Medical records were consulted to collect information on drugs used and falls experienced during the year studied.

The instruments were applied between March and November 2017 by four nurses specialized in rehabilitation nursing, two from each institution, who received information on the objective of the study and examined the use of the instruments. Data collection was carried out using interviews in the case of elderly people without cognitive decline and through observation by nurses in the case of elderly people with cognitive decline. SSPS version 23.0 software was used for statistical analysis.

The Mann-Whitney test was applied as an alternative to the *t*-test for two independent samples, and the Chi-square test was used to analyze the relationship between two qualitative variables. A significance level of p≤0.05 was adopted.^10^

The present study is part of the project “Management of fall risks in devices for elderly people” and was approved by the local ethics committee. The anonymity of the participants and the confidentiality of the data were protected.

## RESULTS

Of the sample of 204 elderly people, 50% had cognitive decline, of which 26.5% were men and 73.5% women. In the group without cognitive decline, 31.4% were men and 68.6% women. Both groups revealed a prevalence of individuals older than 85 years old (65.7% in the group without cognitive decline and 69.6% in the group with cognitive decline). None of the differences had statistical significance (p>0.05).

The length of institutionalization did not differ significantly between the elderly groups with and without decline (p>0.05), but on average, those with cognitive decline were institutionalized for a slightly longer period (43.2±41.4 months), compared with those without cognitive decline (43.1±43.0 months).

Regarding fall episodes, 40.2% of the elderly with cognitive decline experienced at least one fall during the year studied. Fall prevalence among the elderly without cognitive decline was 42.2%, a difference that was not statistically significant (p>0.05).

The TUGT results revealed that only 15.8% of the participants could execute the test in less than 12 seconds, while 47.1% needed more time to perform it, and 37.1% were incapable of doing the test. Analysis of test differences between the elderly groups with and without dementia reached statistical significance (p<0.001), where 68.6% of participants with dementia were incapable of performing the activities of the test, and 31.4% able to execute them in less than 12 seconds. By contrast, of the 102 elderly without cognitive decline, 37.3% were incapable of performing the test and 62.7% able to complete it in less than 12 seconds.

The analysis of the association between fall data and TUGT results showed that 81.4% of the elderly without cognitive decline and 43.9% of those with cognitive decline who fell needed more than 12 seconds to complete the test. The differences were statistically significant in both groups.

Most of the sample were on a polymedication regimen (93.1%), including four or more different medicines per day; but there were no statistically significant differences between the elderly with and without cognitive decline (p>0.05). However, comparison of the number of participants who took one or more benzodiazepines revealed that there were more elderly with cognitive decline (49%) who used this type of medication than without cognitive decline (24.8%), where this difference was statistically significant (p<0.001).

Concerning fall episodes, 40.2% of the elderly with cognitive decline experienced at least one fall during the year, whereas participants without cognitive decline had a fall prevalence of 42.2%. These differences were not statistically significant (p>0.05).

Analysis of the use of benzodiazepines and of falls in the elderly groups with and without cognitive decline demonstrated that falls were associated with the use of this type of medication only for elderly people with cognitive decline (65.9%) (p<0.05). Among the participants who fell, 32.2% used this class of medication.

The evaluation of practices and behaviors to prevent falls using the EPCIPQ indicated that the elderly with cognitive decline had, on average, better safety practices, almost all of which had statistical significance (p<0.001), as shown in Table 1.

**Table 1:** Sample characteristics regarding practices and behaviors to prevent falls among the elderly with and without cognitive decline. Lisbon, Portugal, 2018 (N=204).

Practices and behaviors	Elderly with cognitive decline				Elderly without cognitive decline			P
	Mean	Median	SD		Mean	Median	SD	
1. Tries to be perseverant when choosing the best fall preventive measures	2.83	3.00	.375		2.13	2.00	.691	.000
2. Selects adequate shoes for their feet	2.90	3.00	.299		2.11	2.00	.737	.000
3. Opts to wear closed shoes	2.95	3.00	.217		2.19	2.00	.775	.000
4. Opts to wear shoes with non-skid soles	2.59	3.00	.569		1.78	2.00	.645	.000
5. Organizes room's space to facilitate moving around it	2.59	3.00	.569		1.85	2.00	.702	.000
6. Removes obstacles that might interfere walking in the room	2.91	3.00	.285		2.48	3.00	.627	.000
7. Keeps the bed wheels locked	3.00	3.00	.000		2.70	3.00	.522	.000
8. When getting out of bed, sits at first, with feet touching the ground and stands up after	3.00	3.00	.000		2.85	3.00	.386	.000
9. Makes sure that feet are well supported on the ground before standing up	2.89	3.00	.312		2.71	3.00	.456	.001
10. Verifies if the bathroom floor is not slippery/wet before using it	2.83	3.00	.375		2.72	3.00	.451	.054
11. Makes sure the floor is not slippery before hygiene care	2.95	3.00	.217		2.71	3.00	.537	.000
Total practices and behaviors	2.38	2.36	.38		2.85	3.00	.21	.000

Application of the Mann-Whitney U-test allowed exploration of the hypothesis of existence of differences between the practices of the elderly with and without cognitive decline as a function of gender, age, benzodiazepine use, use of four or more medications, and fall occurrence (Table 2).

**Table 2:** Results of the application of the Mann-Whitney U-test regarding practices and behaviors of elderly with and without cognitive decline. Lisbon, Portugal, 2018 (N=204).

Variable	Cognitive status	Gender	Mean rank	U	Z	P
Gender	Without decline	M	44.05	856.500	-1.461	.144
		F	53.26			
	With decline	M	57.63	847.000	-1.449	.147
		F	49.29			
Use of benzodiazepines	Without decline	Yes	53.62	834.500	-.732	.464
		No	48.78			
	With decline	Yes	49.51	1200.500	-.79	.442
		No	53.41			
Takes four or more medications	Without decline	Yes	51.08	178.500	-1.480	.139
		No	33.35			
	With decline	Yes	52.19	311.500	-.927	.354
		No	43.44			
Falls	Without decline	Yes	44.70	974.500	-1.582	.114
		No	53.90			
	With decline	Yes	46.00	1025.000	-1.776	.076
		No	55.20			
Age	Without decline	≤84	54.24	995.000	-.928	.354
		≥85	48.58			
	With decline	≤84	44.52	884.000	-1.818	.069
		≥85	54.55			

## DISCUSSION

Increasing longevity and prevalence of cognitive alterations makes it more likely that people will be institutionalized. While falls are harmful to elderly living in the community, studies indicate that in long-stay institutions for elderly people, not only is fall incidence higher, but also the resulting injuries are more serious and have a greater negative impact on elderly people’s functioning.^2^^,^ 5 ^,^ 7 The results of the present investigation confirm the high prevalence of falls, given that 41.4% of the elderly examined fell at least once during the course of one year.

The elderly with cognitive decline fell more often, corroborating the results of other studies reporting similar conclusions. An investigation carried out in long-stay institutions for elderly found that 90% of the participants scored < 24 points on the MMSE, and that fall risk increased 5% for each point lower on the test.^5^ Alterations and deficits caused by cognitive decline lead to loss of functional capacity, with reduction and/or loss of abilities, significantly interfering with activities of daily living^11^ and gait, which directly or indirectly increases fall risk.

Among elderly people with cognitive decline, falls are associated with the use of benzodiazepines, whose prolonged use is associated with sedation, amnesia, cognitive deterioration, ataxia, and a higher number of fall episodes.^12^ The proportion of the present sample that took benzodiazepines was 49% among the elderly with cognitive decline and 24.8% among those without cognitive decline, in agreement with the results described in other investigations that have warned about excess use of this drug among institutionalized elderly people (37.5%).^2^

Although there were no significant associations between taking four or more medications per day and occurrence of falls, it was noteworthy that 93.1% of the elderly examined took several medications, whose interactions may increase fall risk. One study revealed that 52% of institutionalized elderly people take eight or more different medications.^2^

Evaluation of mobility revealed that only 15.8% of the elderly assessed could complete the TUGT in less than 12 seconds, 47.1% needed longer, and 37.1% were incapable of performing it. Among institutionalized or frail elderly, the execution time for this test ranges from 10 to 32.6 seconds.^8^^,^ 13

It was concluded that there were associations between performance on the test and fall prevalence in elderly people with or without cognitive decline. It was found that 81.4% of the elderly without cognitive decline and 43.9% of those with cognitive decline who fell took longer than 12 seconds to perform the test, where this difference between the groups was statistically significant.

Future studies should be designed investigating the relationship between falls and acute confusion in institutionalized elderly^14^ and explore the relationship between these variables and length of institutionalization.

Other investigations have demonstrated that elderly who experienced a fall had poorer performance in mobility and gait (TUGT and gait time, 6 meters) and balance (one-leg standing test; tandem walking test) compared to elderly who did not have a fall.^15^ Some researchers have emphasized that gait difficulties associated with the incorrect use of gait assistance devices is one of the main causes of falls,^5^ as well as difficulties moving from one place to another, for instance, transferring from a bed to a chair or from a chair to the toilet.^2^^,^ 7

A study of elderly people with dementia confirmed that falls were more frequent in this group compared to their healthy counterparts.^16^ The authors stressed that, regarding the association between falls and quantitative gait parameters, poorer performance was associated with falls only in healthy elderly people, suggesting that other risk factors contribute to the occurrence of falls in elderly people with dementia.^16^

Gait and balance alterations, in addition to increasing fall risk, may lead to limitations in self-care and increase fear of falling, an aspect that was not explored in the present study because the validated instruments applied can only be used in a population without cognitive decline. Nevertheless, it is essential to question the impact of falls and the fear of further accidents on physical and mental health, socialization, and consequent reduction of mobility and physical fitness, increasing fall risk and impacting costs and the organization of health systems and services.^17^

Regarding safety practices and behaviors, elderly people with cognitive decline were those who generally had the best practices. Reduction in the ability to carry out activities of daily living and instrumental tasks results in less independence and autonomy, lower quality of life, and increases the chances of falling.^18^ This may explain the differences in the practices of people with and without cognitive decline.

Other investigations should explore the reasons underlying these differences. Psychological trauma in elderly people who fall may lead to restriction of activities as a consequence of insecurity regarding the ability to maintain balance, low self-efficacy or self-confidence in preventing falls, and anticipation of the potential consequences of a fall episode.^19^

Because falls in institutionalized elderly people are associated with location, occurring mostly in bedrooms (30%), while walking (37.1%), and when getting out of bed (25.7%),^2^ the authors consider that the practices adopted by most elderly people with cognitive decline are positive, such as keeping the bed wheels locked, using the proper technique when getting out of bed, and selecting closed, non-skid shoes.

The present study has some limitations: the sample (sample size and sampling technique); the adoption of two different ways of collecting data; and the use of retrospective fall records, which are not considered the best approach to evaluate falls because of the risk of unreported fall episodes, especially in elderly people with cognitive decline, resulting in underreporting of the actual number of falls.

Despite these limitations, the data obtained in the present study contribute to better understanding of risk factors in the genesis of falls and of practices and behaviors of elderly people with and without cognitive decline to maintain patient safety during self-care and access to the physical spaces.

In conclusion, the authors stress that, although the practices and behaviors of elderly people with cognitive decline were safer, on average, compared to those of elderly people without cognitive decline, the former had a higher fall prevalence, which was associated with time taken to execute the TUGT and use of benzodiazepines. No relationships were found between gender, age and length of institutionalization, and the practices and behaviors, and fall in either group. Due to the complexity of the factors involved in the genesis of fall risks and fall episodes and the lack of knowledge of risk factors in institutionalized people with cognitive decline, the authors agree with other researchers in advocating the introduction of interventions to prevent falls in people who score lower than 24 points on the MMSE.^5^
